# Fe_3_O_4_ Nanoparticles on 3D Porous Carbon Skeleton Derived from Rape Pollen for High-Performance Li-Ion Capacitors

**DOI:** 10.3390/nano11123355

**Published:** 2021-12-10

**Authors:** Mingshan Sun, Xinan Chen, Shutian Tan, Ying He, Petr Saha, Qilin Cheng

**Affiliations:** 1 Key Laboratory for Ultrafine Materials of Ministry of Education, School of Materials Science and Engineering, East China University of Science and Technology, Shanghai 200237, China; minsen.sun@foxmail.com (M.S.); y30190460@mail.ecust.edu.cn (X.C.); y30190527@mail.ecust.edu.cn (S.T.); 2 Sino-EU Joint Laboratory of New Energy Materials and Devices, Tomas Bata University in Zlin, nam. T. G. Masaryka 5555, 760 01 Zlin, Czech Republic; saha@utb.cz

**Keywords:** Fe_3_O_4_, porous carbon, Li-ion capacitor, electrochemical properties

## Abstract

Herein, a three-dimensional (3D) Fe_3_O_4_@C composite with hollow porous structure is prepared by simple solution method and calcination treatment with biomass waste rape pollen (RP) as a carbon source, which is served as an anode of Li-ion capacitor (LIC). The 3D interconnected porous structure and conductive networks facilitate the transfer of ion/electron and accommodate the volume changes of Fe_3_O_4_ during the electrochemical reaction process, which leads to the excellent performance of the Fe_3_O_4_@C composite electrode. The electrochemical analysis demonstrates that the hybrid LIC fabricated with Fe_3_O_4_@C as the anode and activated carbon (AC) as the cathode can operate at a voltage of 4.0 V and exhibit a high energy density of 140.6 Wh kg^−1^ at 200 W kg^−1^ (52.8 Wh kg^−1^ at 10 kW kg^−1^), along with excellent cycling stability, with a capacity retention of 83.3% over 6000 cycles. Hence, these encouraging results indicate that Fe_3_O_4_@C has great potential in developing advanced LICs electrode materials for the next generation of energy storage systems.

## 1. Introduction

As the limited resources of crude oil and deteriorating ecological environment, especially the greenhouse effect and discharge of poisonous substances, it is particularly urgent to explore and develop a green source of sustainable energy [1,2]. Supercapacitors (SCs) and lithium-ion batteries (LIBs) have increasingly become a study focus regarding electrochemical energy storage systems in recent decades [3]. None of these stand-alone technologies, however, can meet today’s commercial demands for both energy and power [4,5,6]. As a result, it is crucial to make high energy and high power, together with extended cycling life run concurrently in an integrated high-efficiency energy storage system [7,8]. In response to this demand, lithium-ion capacitors (LICs), which can well combine the rapid power output of SCs and the high energy density of LIBs, would be highly promising, competitive, and reliable candidates for future energy-storage devices [9].

However, the serious bottleneck problem restricting the application of LICs is the imbalanced kinetics that occurs on the cathode and anode [10,11,12,13]. With this in mind, the fabrication of electrode materials with excellent electrochemical properties is an effective approach to ameliorate this issue [12,14]. As is well known, Fe_3_O_4_, as the anode of LIBs, has attracted intensive attention in the past consecutive years because of its cost effectiveness, eco-friendliness, low intercalation potential, and high theoretical reversible capacity [15,16,17,18,19,20]. Nevertheless, there are still some disadvantages that result in poor electrochemical performance of Fe_3_O_4_, which has limited its practical application to a large extent. On the one hand, the large volume change (~93%) of pure Fe_3_O_4_ during electrochemical reaction gives rise to the crushing and electrical contact loss of electrode materials [15,18,21]. On the other hand, the low inherent conductivity of Fe_3_O_4_ also weakens its rate performance [22]. For the sake of improvement of the defect in the electrochemical kinetics of Fe_3_O_4_, the easiest and most effective approach is to introduce carbonaceous materials into the system by the design of anode electrode materials with suitable nanoarchitectures to prevent agglomeration and enhance their electronic conductivity [19,23].

In recent years, electrode materials with novel structures have been designed by using natural organic biomass such as microalgae [24], lychee exocarp [25], corncob [26], and peanut shells [8] as carbon sources. Rape pollen (RP) grains have aroused great interest for their characteristic 3D porous architecture [27,28]. The structure of this hollow sphere can optimize the volumetric expansion of electrode material and also bridge the diffusion path of Li-ions owing to the abundant cavities inside the hollow sphere.

Herein, a new anode material of LIBs was designed and fabricated, in which Fe_3_O_4_ nanoparticles were uniformly anchored on the RP-derived 3D hollow porous carbon skeleton by pyrolyzing RP grain together with ferric chloride and sodium fluoride. As expected, the composite exhibits better electrochemical performance than the 3D hollow biochar or Fe_3_O_4_ nanoparticles alone. The resulting porous Fe_3_O_4_@C composite electrode demonstrates a high reversible capacity of 918 mAh g^−1^ at 100 mA g^−1^ as well as superior rate capability (719 mAh g^−1^ even at 2 A g^−1^). Furthermore, the hybrid LIC fabricated with Fe_3_O_4_@C composite as anode and activated carbon (AC) as cathode achieves a high energy density of 140.6 Wh kg^−1^ at 200 W kg^−1^, and 52.8 Wh kg^−1^ even at an ultrahigh power density of 10 kW kg^−1^.

## 2. Materials and Methods

### 2.1. Pretreatment and Carbonization of Rape Pollen (RP)

The RP grains were purchased from Wangs Co. Ltd. Initially, in order to remove the surface impurities and proteins in the core, the RP grains were thoroughly ground and soaked into ethanol for one week. After filtration and washing with ethanol and distilled water, the samples were then freeze dried at −50 °C for 12 h. Afterward, the pretreated RP grains were directly pyrolyzed in an oven at 700 °C for 3 h in a N_2_ atmosphere. The resulting black powder was washed with 1 M HCl and deionized water, followed by drying for 24 h at −50 °C in a freeze dryer.

### 2.2. Fabrication of Fe_3_O_4_@C Hollow Porous Composite

Firstly, the black RP-derived carbon sample was treated with nitric acid to obtain oxygen-rich functional groups (carboxyl and hydroxyl groups) on the surface. Subsequently, the hydrolysis method was employed to in situ grow Fe_3_O_4_ on the functionalized porous carbon. Typically, 0.05 g C powder was stably suspended in 150 mL deionized water upon ultrasonication for 1 h. Then, 0.54 g FeCl_3_ and 0.35 g NaF were put into the above suspension, and the reaction was kept at 90 °C for 24 h with continuous stirring. Later, the precipitate was obtained by centrifugation and freeze drying. The yellow product was followed by annealing at 600 °C for 4 h in N_2_, and the porous Fe_3_O_4_@C powder sample was thus collected.

### 2.3. Material Characterization

The crystal structures of the Fe_3_O_4_@C nanocomposite and pure C sample were studied by a powder X-ray diffractometer (XRD, RIGAKU, D/MAX 2550 VB/PC, Tokyo, Japan) at room temperature. X-ray photoelectron spectroscopy (XPS) was carried out on an ESCALab 250Xi spectrometer (Waltham, MA, USA) with an Al Kα source and the C 1s peak as the internal standard at 284.8 eV. The morphologies and texture of the samples were examined by field emission scanning electron microscopy (FE-SEM, Hitachi S-4800, Tokyo, Japan), and the element distribution was investigated with an energy-dispersive spectroscopy (EDS) detector (HITACHI, Tokyo, Japan). Raman spectra were conducted on an Invia Micro-Raman spectrometer (Renishaw, London, United Kingdom) with an excitation wavelength of 532 nm. Thermogravimetric measurements (TGA, NETZSCH TG209F1, Selb, Germany) were employed to determine the loading of carbon in Fe_3_O_4_@C in air.

### 2.4. Fabrication and Evaluation of the Half Cells and LICs

In order to prepare the working electrode, a mixture of 80 wt% Fe_3_O_4_@C powder, 10 wt% acetylene black, and 10 wt% polyvinylidene fluoride in N-methyl-2-pyrrolidone (NMP) was formed homogeneously into a slurry, which was then uniformly cast on a copper foil and vacuum dried at 120 °C for 12 h. The electrochemical performances were investigated in CR2016 coin cells that were packaged in a glove box full of argon. For half-cell configuration, both counter and reference electrode were a pure lithium metal, while the separator and electrolyte used were a Celgard 2400 membrane and 1 M LiPF_6_ dissolved in co-solvents of ethylene carbonate/diethyl carbonate/dimethyl carbonate (*v*/*v*/*v*, 1:1:1). The AC electrode was also made similar to the above steps. The AC powder, acetylene black, and PVDF (7:2:1, weight ratio) were mixed in NMP to form a paste and coated on an Al foil, followed by vacuum drying at 120 °C for 12 h.

To fabricate Fe_3_O_4_@C//AC LICs, the prelithiated Fe_3_O_4_@C anode and AC cathode were assembled in CR2032 coin cells. In order to make up for the Li^+^ loss from the first anode discharge, Fe_3_O_4_@C anode was prelithiated with Li plate as the reference electrode before assembling the device. The prelithiation process was carried out by discharging and charging for 5–10 cycles at 0.1 A g^−1^ within 0.01–3.0 V and then finished with the voltage of lithiation down to 0.01 V. The mass ratio between cathode and anode was controlled to 2:1, 3:1, 4:1, 5:1, respectively. The electrolyte and separator were the same as above.

### 2.5. Electrochemical Measurements

Cyclic voltammetry (CV) and electrochemical impedance spectroscopy (EIS) were performed on an electrochemical workstation (CHI660E), but the galvanostatic charge–discharge (GCD) performance was evaluated using a battery test instrument (Land CT2001A) at room temperature. The specific capacitances of half-cells and LICs are derived from the following equation:

*C* = *I*/[(dV/dt) × *m*] ≈ *I*/[(△*V*/△*t*) × *m*] (F g^−1^), (1)

where *I* is the constant discharge current, ∆*V* denotes the total potential window, ∆t indicates the full discharge time, and *m* represents the mass of the active material. The energy density (*E*) of LICs is defined by the following formula:

*E* = *CV*^2^/2 × 3.6(Wh kg^−1^), (2)

The power density (*P*) of LICs is calculated by the energy density (E), and the discharge time (*t*) based on the following equation:

*P* = *E* × 3600/*t* (W kg^−1^) (3)

## 3. Results and Discussion 

**Scheme 1:**
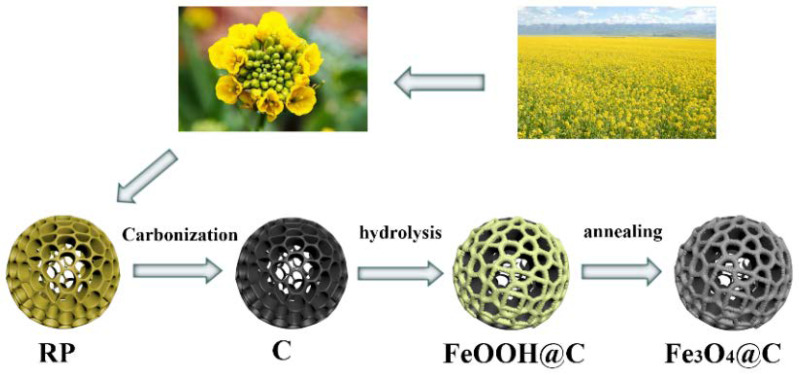
The preparation process of porous Fe_3_O_4_@C composite.

Scheme 1 illustrates the preparation procedure of a 3D porous Fe_3_O_4_@C composite. Firstly, RP grains were pretreated with ethanol to remove impurities and some inside components. Then, they were calcined under nitrogen protection to convert to a porous carbon skeleton. Subsequently, the black RP-derived carbon was further treated with HNO_3_ to make its surface negatively charged, which is in favor of ferric ion (Fe^3+^) adsorption. Driven by electrostatic attraction, Fe^3^ was adsorbed onto porous carbon and gradually changed into FeOOH particles via a chemical bath deposition method, followed by an annealing process in nitrogen; the 3D porous Fe_3_O_4_@C was thus achieved. 

**Figure 1:**
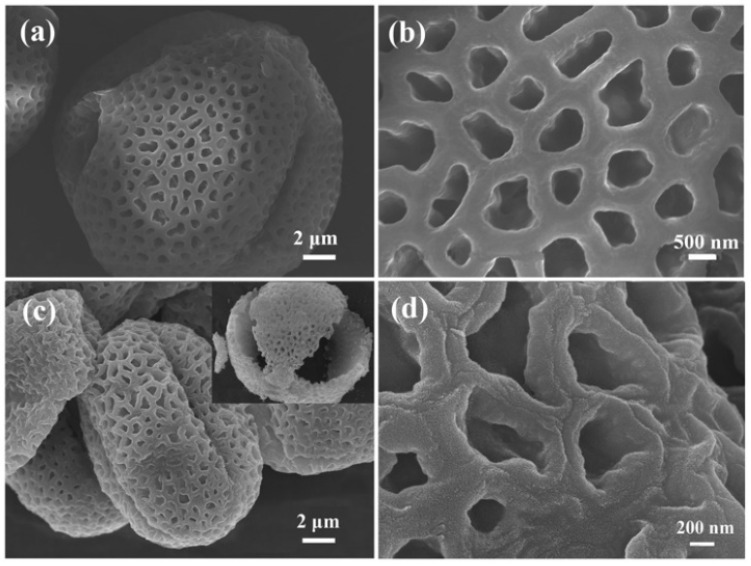
SEM images of RP (**a**,**b**) and RP-derived C (**c**,**d**) at low and high magnification.

The detailed morphology and microstructure were analyzed by SEM. The typical images of RP grains and RP-derived porous carbon are presented in Figure 1. Figure 1a clearly indicates that the natural RP grain after pretreatment exhibits an ellipsoidal morphology with a 3D porous structure and a long axis of about 15 μm. The magnified image further reveals many micro/nanocavities with a size of less than 1μm on the RP surface (Figure 1b). Apparently, the RP-derived carbon particles suffer shrinkage of the structure to some extent after the carbonization process but still remain the elliptical shape with porous networks (Figure 1c). Significantly, the surface of RP-derived carbon becomes rough after acid treatment due to rich carboxyl and hydroxyl groups (Figure 1d). This change makes it more susceptible for FeOOH to grow near the surface of porous carbon, which is caused by electrostatic adsorption between iron ions and negatively charged surface groups. 

**Figure 2:**
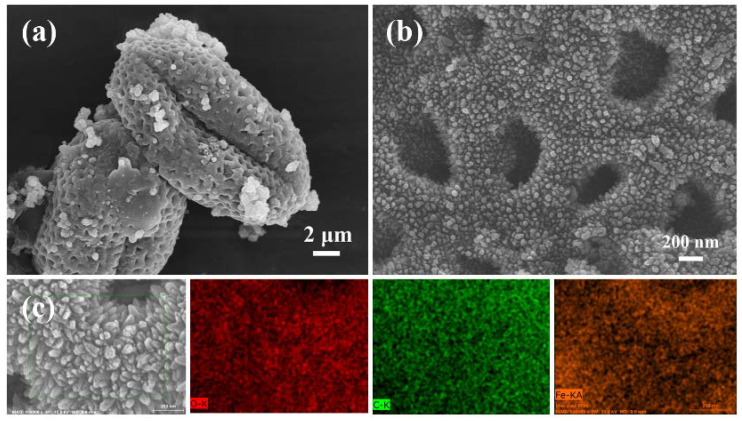
(**a**,**b**) SEM images of Fe_3_O_4_@C at low and high magnification; (**c**) EDS elemental mapping image (**c**) of Fe_3_O_4_@C.

The target product Fe_3_O_4_@C was subsequently obtained by heat treatment of FeOOH@C composite. As illustrated in Figure 2a,b, the as-prepared Fe_3_O_4_@C consists of porous carbon networks with plenty of Fe_3_O_4_ nanoparticles densely decorated throughout the surface. The EDS mapping of a single Fe_3_O_4_@C sphere reveals the homogeneous distribution of Fe, O, and C elements in the composite (Figure 2c), which indicates that Fe_3_O_4_ nanoparticles are successfully and uniformly deposited on RP-derived carbon. In addition, the composite maintains the original ellipsoidal morphological characteristics of RP, suggesting that the pyrolysis process does not destroy the microstructure of the carbon skeleton. Such a unique 3D interconnected carbon framework provides ample void spaces to relieve volume changes of Fe_3_O_4_ during the lithiation process and also offers plenty of active regions for the electrochemical reaction of Fe_3_O_4_ and promoting electrolyte penetration and lithium-ion diffusion processes. The pore structure of Fe_3_O_4_@C composite can be confirmed by N_2_ adsorption/desorption isotherms shown in Figure S1. An obvious hysteresis loop at a relative pressure of 0.4–0.9 indicates the presence of mesopores, while the dramatic nitrogen uptake at a high pressure of 0.9–1.0 suggests the macropores feature in the composite. The corresponding pore size distribution reveals the dominated pore size is in the range of 7.9–10.8 nm. Hence, the mesopores in the composite benefit the transport of electrolyte ions, and the macropores serve as ion-buffering reservoirs to shorten the diffusion distance [29], which thus boosts the Li storage performance of Fe_3_O_4_@C anode.

**Figure 3:**
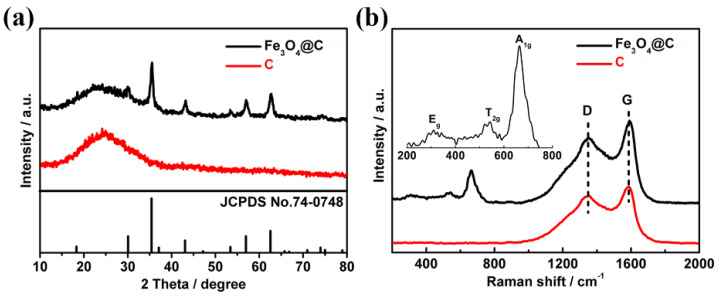
(**a**) XRD patterns of Fe_3_O_4_@C composite, RP-derived carbon; (**b**) Raman spectra of Fe_3_O_4_@C composite and RP-derived carbon. The inset shows the enlarged region at low Raman shift.

The XRD patterns of RP-derived carbon and Fe_3_O_4_@C composite formed by carbothermal reduction of iron hydroxide (Figure S2) at 600 °C are given in Figure 3a. A wide diffraction peak at about 24.8° in both samples can be indexed as the typical amorphous carbon structure of RP-derived carbon. Moreover, the Fe_3_O_4_@C composite also displays distinct characteristic peaks at 18.2°, 30.1°, 35.4°, 43.2°, 53.4°, 57.1°, and 63°; all of those peaks well correspond to the (111), (220), (311), (400), (422), (333) and (440) lattice planes of typical Fe_3_O_4_ (JCPDS NO. 74-0748), respectively. It is worth noting that the grain size of Fe_3_O_4_ particles on porous carbon is calculated to be 12.6 nm for the (311) diffractions by Scherrer’s equation, which is smaller than that shown in Figure 2b. This is mainly caused by the agglomeration of particles during the annealing process. Figure 3b shows the Raman spectra of both samples. It is evident that both Fe_3_O_4_@C and carbon exhibit two peaks at 1349 and 1591 cm^−1^ corresponding to D and G bands of carbon, and the intensity ratio (ID/IG) of the D band to the G band was calculated to be 0.97, suggesting the disordered and sp^2^ graphitic structure, which can lead to more Li^+^ storage active sites for redox reactions [30]. Furthermore, the characteristic peaks of Fe_3_O_4_ are also clearly detected at 663 cm^−1^ (A_1g_), 542 cm^−1^ (T_2g_), and 309 cm^−1^ (E_g_) in the XRD pattern of the Fe_3_O_4_@C sample, further confirming the formation of the spinel Fe_3_O_4_ phase [31]. Thus, the results of Raman and XRD reveal the successful growth of Fe_3_O_4_ on the carbon skeletons. In addition, in order to determine the proportion of Fe_3_O_4_ in the composite, TGA was performed on Fe_3_O_4_@C and carbon in an air atmosphere (Figure S3). A slight increase in the weight occurs before the mass loss, which is due to the inevitable oxidation of a small amount of Fe_3_O_4_ at high temperature, but a significant weight loss at 480 °C–550 °C is attributed to the transformation of C into CO_2._ Thus, the loading of Fe_3_O_4_ in Fe_3_O_4_@C is calculated to be around 83.7 wt%.

**Figure 4:**
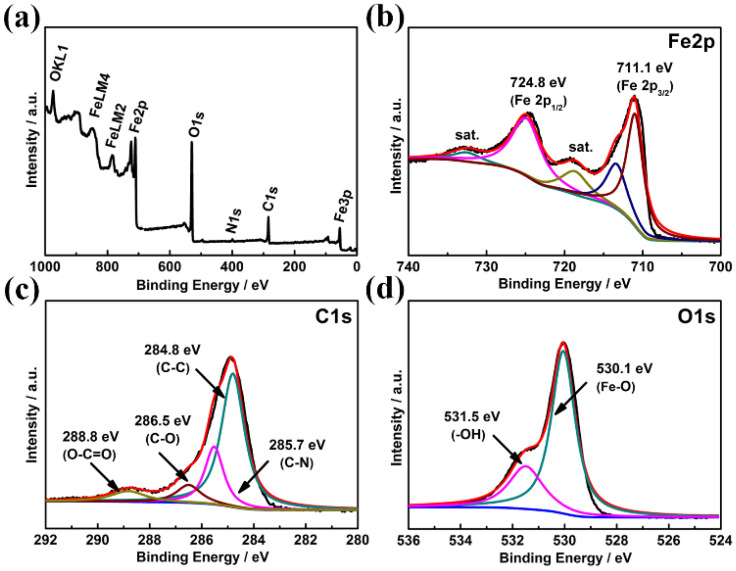
(**a**) wide survey, (**b**) Fe 2p, (**c**) O 1s, and (**d**) C 1s of XPS spectra of Fe_3_O_4_@C.

To further analyze the surface composition of Fe_3_O_4_@C composite, XPS measurements were carried out (Figure 4). The wide survey spectrum of Fe_3_O_4_@C composite (Figure 4a) demonstrates only four elements in the composite: C, O, Fe, and N, reflecting the high purity of the sample. The small amount of N-doping results from the carbonization of RP grains, which contributes to the surface wettability and conductivity of the composite. The high-resolution Fe 2p spectrum (Figure 4b) indicates that the two peaks at 711.1 eV and 724.8 eV are assigned as the spin-orbit peaks of Fe 2p^3/2^ and Fe 2p^1/2^, respectively. In addition, the characteristic satellite peak of 719.3 eV further illustrates the presence of Fe_3_O_4_ [32]. In Figure 4c, the C 1s spectrum of Fe_3_O_4_@C appears a peak with a maximum intensity at 284.8 eV, which represents the C−C bond. The peak centered at 285.7 eV is associated with t the C−N bond. Furthermore, the two peaks at 286.5 and 288.8 eV correspond to C−O and O−C=O bonds, respectively. Additionally, the O 1s spectrum of Fe_3_O_4_@C is given in Figure 4d. Specifically, the extreme point at 530.1 eV represents a typical Fe-O bond [33].

**Figure 5:**
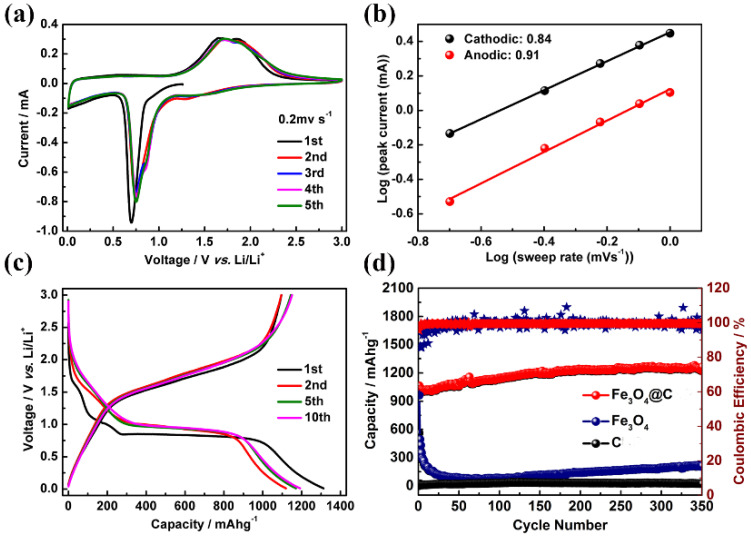
(**a**) The first five CV curves of Fe_3_O_4_@C electrode at a scan rate of 0.2 mV s^−1^; (**b**) *b* value determined based on the dependence of the peak current on the scan rate; (**c**) GCD profiles of Fe_3_O_4_@C electrode at 0.1 A g^−1^; (**d**) cycling stability and CE of different electrodes at 0.1 A g^−1^.

To characterize the electrochemical behavior of the Fe_3_O_4_@C electrode, various electrochemical measurements were performed. Figure 5a displays the first five CV curves for Fe_3_O_4_@C anode at 0.2 mV s^−1^. One obvious sharp peak in the first cycle observed at ~0.71 V during the cathodic process is caused by the transformation of Fe^3+^/Fe^2+^ to Fe (0) and the occurrence of solid electrolyte interphases (SEI) [34]. As for the next four cycles, the peaks shift to a higher voltage of 0.76 V and almost remain consistent, suggesting the stability of the SEI films without any other side reactions except for the conversion of Fe^3+^/Fe^2+^ into Fe (0). Moreover, the intensity of the reduction current in the first cycle is stronger, which further demonstrates the occurrence of irreversible reactions. Both anodic peaks at about 1.65 and 1.85 V can be originated from the oxidation of the iron metal to Fe_3_O_4_. To reveal the kinetics behavior of the Fe_3_O_4_@C electrode, Figure S4 presents the CV curves at various scanning rates. As a rule, the relationship between the peak current (*i*) and scanning rate (*v*) follows the power law [35].

*i* = a*v*^b^, (4)

log (*i*) = *b* log (*v*) + log *a*, (5)

where both *a* and *b* represent constants. The value of *b* can be calculated by the slope of log *i* vs. log *v* during the corresponding anodic and cathodic CV processes shown in Figure S4. As *b* value is 0.5, Li storage is dominated by diffusion-controlled behavior associated with Li^+^ intercalation and transportation, while *b* = 1 denotes a pseudocapacitive-controlled process related to a surface faradaic redox reaction [36,37]. As presented in Figure 5b, the anodic and cathodic peaks of Fe_3_O_4_@C electrode show a good linear correlation under various scan rates, and the obtained *b* values are 0.91 and 0.84, respectively, demonstrating that the fast kinetics is mainly caused by the pseudocapacitive-controlled behavior, and this, in turn, confirms that the Fe_3_O_4_ nanoparticles on the porous carbon networks are beneficial for fast faradic reaction.

Figure 5c depicts the first ten galvanostatic data for Fe_3_O_4_@C electrode at 0.1 A g^−1^. Fe_3_O_4_@C displays a discharge plateau at 0.81 V in the first cycle, and the plateaus move to about 0.89 V in the following nine cycles, which is consistent with the CV results. Simultaneously, the charge curves show plateaus between 1.5 and 2.0 V in line with the CV anodic peaks. Under the initial cycle, the composite electrode provides a discharge capacity of 1312 mAh g^−1^ and a reverse capacity of 1096 mAh g^−1^ with the initial Coulombic efficiency (CE) of 83.5%. Notably, the SEI films formation results in irreversible capacity decay. In the subsequent cycles, the Fe_3_O_4_@C electrode shows better specific capacity and CE, indicating the good reversibility of conversion reaction and structural stability of the composite electrode. Figure 5d illustrates the cycling properties of Fe_3_O_4_@C, Fe_3_O_4,_ and RP-derived carbon electrodes. The capacity of Fe_3_O_4_ decreases rapidly, which may be attributed to the efflorescence of Fe_3_O_4_. In comparison with Fe_3_O_4_, Fe_3_O_4_@C demonstrates more stable cycling performance; its specific capacity decreases initially and increases gradually with the cycle number, indicating a lithium-induced re-activation of Fe_3_O_4_@C electrode, which is probably caused by the pulverization of Fe_3_O_4_ and irreversible reactions with Fe nanoparticles formation during the charge/discharge process. The pulverization can increase the surface area of the electrode in favor of more active sites for Li storage, while Fe particles contribute to the overall conductivity to facilitate the charge transfer kinetics [17,38]. Moreover, the Fe_3_O_4_@C electrode retains a specific capacity of 1262 mA h g^−1^ after 350 cycles, further confirming that the porous carbon networks ensure excellent structural stability of the Fe_3_O_4_@C composite electrode.

**Figure 6:**
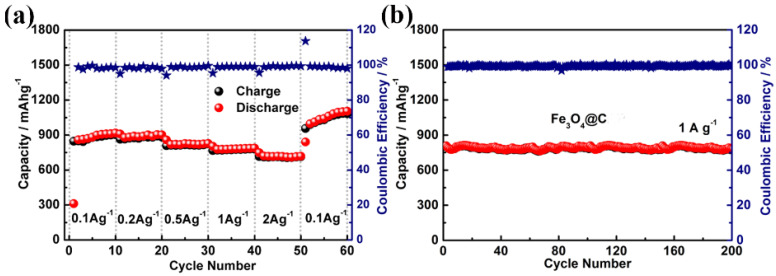
(**a**) Rate capability and CE of Fe_3_O_4_@C electrode at 0.1 to 2 A g^−1^; (**b**) cycling stability and CE of Fe_3_O_4_@C at 1 A g^−1^ for 200 cycles.

To further confirm the high-rate capability of the composite electrode, the cycle charge and discharge tests were performed at 0.1 to 2 A g^−1^, shown in Figure 6a. As the current density is 0.1, 0.2, 0.5, 1, and 2 A g^−1^, the reversible discharge specific capacities are 918, 902, 828, 788, and 719 mAh g^−1^, respectively, while the current density returns to 0.1 A g^−1^, Fe_3_O_4_@C anode rapidly returns to a high capacity of 1083 mAh g^−1^. These phenomena indicate that the Fe_3_O_4_@C has excellent rate capability and structural integrity. The cycle life of the composite electrode at a high current density was also tested at room temperature. Apparently, the composite electrode possesses a high capacity of 780 mAh g^−1^ after 200 cycles at 1.0 A g^−1^, with a retention rate as high as 96.6% (compared with the second discharge capacity), showing excellent cycling stability. The enhanced electrochemical properties of Fe_3_O_4_@C were further confirmed by EIS measurement (Figure S5). As indicated by the Nyquist plots of both electrodes, the spectra consist of a semicircle in a high-frequency region and an inclined line in a low-frequency region. An equivalent circuit by fitting the EIS pattern includes bulk solution resistance (*R*_s_), the charge transfer resistance (*R*_ct_), and the Warburg impedance (*R*_w_) relating to ion diffusion [39]. Clearly, the Fe_3_O_4_@C electrode possesses a much smaller diameter of the semicircle than the pure Fe_3_O_4_ electrode, i.e., very lower *R*_ct_ (27.5 Ω) than Fe_3_O_4_ (415.3 Ω), indicating the interconnected porous carbon networks in composite increase the conductivity and facilitate the charge transfer between Fe_3_O_4_@C and electrolyte during the electrochemical reaction. In addition, a larger slope of the inclined line for Fe_3_O_4_@C implies a lower Li^+^ diffusion resistance (*R*_w_). Therefore, the synergistic effect between porous carbon and Fe_3_O_4_ enables fast transfer of ion/electron at the electrode–electrolyte interface and rapid diffusion rate of electrolyte ions into the electrode, thus leading to better electrochemical performance of Fe_3_O_4_@C electrode.

Full cells of LICs were constructed using AC cathode and Fe_3_O_4_@C anode materials. The electrochemical behavior of AC is also of great importance to the overall performance of LICs, which was first investigated in LIB half-cell ranging from 2.0 to 4.0 V. Notably, the quasi-rectangular CV curves at 2–20 mV s^−1^ exactly verify that EDLC behavior is caused by desorption–adsorption of the PF_6_^−^ anion on the surface of AC electrode (Figure S6a). Meanwhile, a high discharge capacity and a favorable rate capability, together with long cycle life, were also achieved for the AC electrode (Figure S6b,c).

Before assembling the LIC device, the Fe_3_O_4_@C electrode was first pretreated in a half-cell for five cycles. Considering that both cathode and anode electrodes have diverse Li^+^ storage behavior, the fabricated LIC was subjected to CV tests at 100 mV s^−1^ (Figure S7). As a result, 0–4.0 V was selected as the optimal voltage window to explore the properties of LIC devices. On the other hand, because the specific capacity of the anode and cathode does not match, an optimal mass ratio of both electrodes is essential to maximize the energy density and power density of the LIC. Figure S8 presents the Ragone plots of the Fe_3_O_4_@C//AC LIC at different mass ratios of Fe_3_O_4_@C to AC, and the optimal mass ratio of 1:3 was thus determined. Additionally, then, the electrochemical performance of the optimized device was systematically investigated.

**Figure 7:**
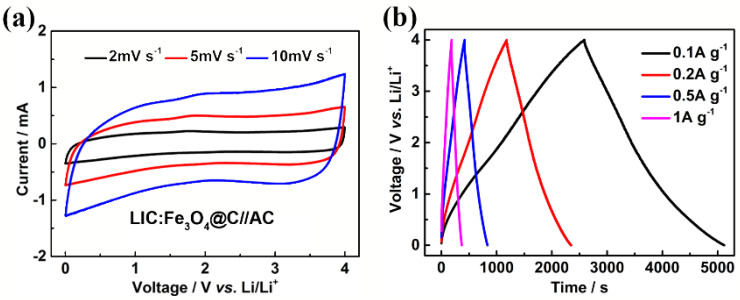
(**a**) CV curves of Fe_3_O_4_@C//AC LIC at 2–10 mV s^−1^, (**b**) GCD profiles of Fe_3_O_4_@C//AC LIC at 0.1–1 A g^−1^.

Figure 7a displays the CV profile of the obtained LIC at 2–10 mV s^−1^. Clearly, all the CV curves show almost the quasi-rectangular shape, demonstrating a favorable dynamics match between the anode and cathode electrodes. Moreover, this LIC device can operate stably over the voltage range from 0 to 4 V. Figure 7b illustrates typical GCD curves for Fe_3_O_4_@C//AC LIC at 0.1–2 A g^−1^, which exhibit a nearly linear and symmetric relationship, demonstrating an effective combination with different charge storage mechanism between both electrodes in this system. 

**Figure 8:**
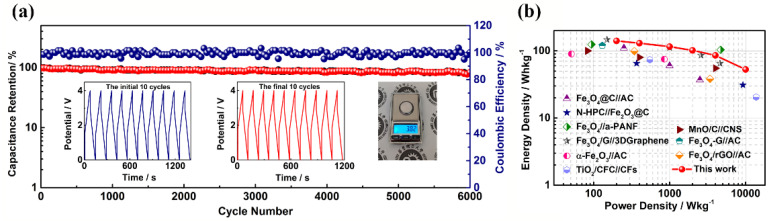
(**a**) Long-term cycling performance and CE of Fe_3_O_4_@C//AC LIC at 2 A g^−1^. Inset: GCD profiles of the initial 10 cycles (blue) and the final 10 cycles (red) and the optical photograph showing the microscale worked by our LIC device; (**b**) Ragone plots of LIC in our work and other previously reported LICs.

To further explore the cycling stability of the Fe_3_O_4_@C//AC LIC device, its cycling performance at a high current density of 2.0 A g^−1^ was also evaluated. As expected, it shows a capacity retention rate of 83.3% after 6000 cycles and a CE of nearly 100% in the whole process (Figure 8a), which is indicative of pretty good long-term stability. The Ragone diagram of the Fe_3_O_4_@C//AC LIC is presented in Figure 8b. This device can deliver a high energy density of 140.6 Wh kg^−1^ at 200 W kg^−1^. Even as the power density is increased to 10 kW kg^−1^, the device still remains 52.8 Wh kg^−1^. Furthermore, the Ragone diagram confirms that our LIC in power/energy combination is superior to the reported LICs such as Fe_3_O_4_@C//AC [40], N-HPC//Fe_2_O_3_@C [41], Fe_3_O_4_//a-PANF [42], Fe_3_O_4_/G//3Dgraphene [43], α-Fe_2_O_3_//AC [44], TiO_2_/CFC//CFS [45], MnO/C//CNS [46], Fe_3_O_4_-G//AC [47], and Fe_3_O_4_/rGO//AC [31], which further indicate that the assembled LIC would have a very strong competitive advantage over the future energy storage systems. The excellent performance of Fe_3_O_4_@C//AC LIC could be attributed to three aspects: (i) RP-derived porous carbon improves the electrical conductivity of Fe_3_O_4_@C composite electrode for rapid charge transfer and offers a large electroactive surface for fast reaction kinetics during charge–discharge process; (ii) 3D porous, interpenetrating carbon networks could ensure uniform growth of Fe_3_O_4_ nanoparticles on the porous carbon networks and efficiently alleviate the volume effect of Fe_3_O_4_ in the electrochemical process, thus endowing Fe_3_O_4_@C with strong structural stability; (iii) the favorable synergistic effect of Fe_3_O_4_ and RP-derived carbon, and good matching of electrode kinetics balance between Fe_3_O_4_@C anode and AC cathode result in significantly enhanced performance of Fe_3_O_4_@C//AC LIC system.

## 4. Conclusions

In summary, Fe_3_O_4_@C composites for the anode of LICs were synthesized using rape pollen as biomass carbon skeleton by a simple, efficient hydrolysis and annealing method. The resulting porous Fe_3_O_4_@C anode delivers a high reversible capacity of 918 mAh g^−1^ at 100 mA g^−1^ and superior rate performance. Furthermore, the hybrid LIC fabricated with Fe_3_O_4_@C anode and AC cathode achieves a high energy density of 140.6 Wh kg^−1^ at 200 W kg^−1^, and an energy density of 52.8 Wh kg^−1^ even at an ultrahigh power density of 10 kW kg^−1^. Moreover, this device can keep over 80% of the capacity over 6000 cycles. Our studies offer a new way for the exploration and fabrication of next-generation energy-storage devices.

## Data Availability

Summary data available upon request.

## Supplementary Materials

The following are available online at https://www.mdpi.com/article/10.3390/nano11123355/s1, Figure S1: Nitrogen adsorption–desorption isotherms of Fe_3_O_4_@C composite. Inset: Pore size distributions of Fe_3_O_4_@C composite, Figure S2: XRD pattern of FeOOH@C composite and the standard XRD pattern of FeOOH, Figure S3: TGA curves of (a) rape pollen recorded in N_2_, (b) C, and Fe_3_O_4_@C composite recorded in air, Figure S4: CV curves of Fe_3_O_4_@C electrode at 0.2–1 mV s^−1^, Figure S5: Nyquist plots of Fe_3_O_4_ and Fe_3_O_4_@C electrodes. Inset: Enlarged plot of high-frequency range and the equivalent electrical circuit used to fit the experimental impedance spectra of Fe_3_O_4_@C electrode, Figure S6: (a) CV curves of AC electrode at 2–20 mV s^−1^; (b) GCD profiles at 0.2–6.4 A g^−1^; (c) the cycling performance of AC at 0.2 A g^−1^ between 2.0 and 4.0 V, Figure S7: The CV curves of Fe_3_O_4_@C//AC LIC at a scan rate of 100 mV s^−1^, Figure S8: Ragone plots of the Fe_3_O_4_@C//AC LIC with mass ratios from 1:2 to 1:5.
