# Supplementary material for: Fe3O4 Nanoparticles on 3D Porous Carbon Skeleton Derived from Rape Pollen for High-Performance Li-Ion Capacitors
Source: Nanomaterials (Basel). 2021 Dec 10;11(12):3355. doi: 10.3390/nano11123355 (PMC8707608; doi:10.3390/nano11123355)
Supplement: Supplementary file 1 [file nanomaterials-11-03355-s001.zip › nanomaterials-1462364-supplementary.pdf]

Supplementary Materials

Fe_3O_4 Nanoparticles on 3D Porous Carbon Skeleton Derived from Rape Pollen for High-Performance Li-Ion Capacitors

Mingshan Sun [1], Xinan Chen [1], Shutian Tan [1], Ying He [1,2,*], Petr Saha [2], and Qilin Cheng [1,2,*]

[1] Key Laboratory for Ultrafine Materials of Ministry of Education, School of Materials Science and
Engineering, East China University of Science and Technology, Shanghai 200237, China;
minsen.sun@foxmail.com (M.S.); y30190460@mail.ecust.edu.cn (X.C.); y30190527@mail.ecust.edu.cn (S.T.)

[2] Sino-EU Joint Laboratory of New Energy Materials and Devices, Tomas Bata University in Zlin, nam. T. G. Masaryka 5555, 760 01 Zlin, Czech Republic; saha@utb.cz

* Correspondence: rehey@ecust.edu.cn (Y.H.); chengql@ecust.edu.cn (Q.C.)

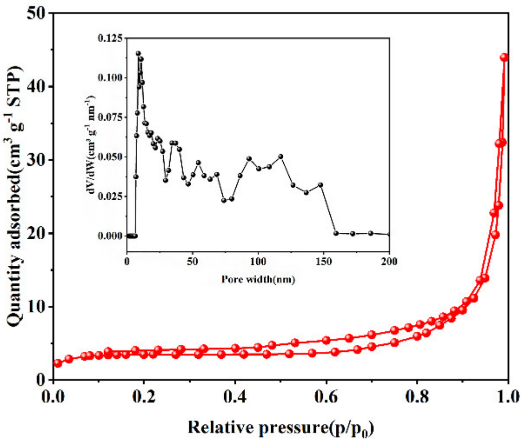

Figure S1. Nitrogen adsorption-desorption isotherms of Fe_3O_4@C composite. Inset: Pore size distributions of Fe_3O_4@C composite.

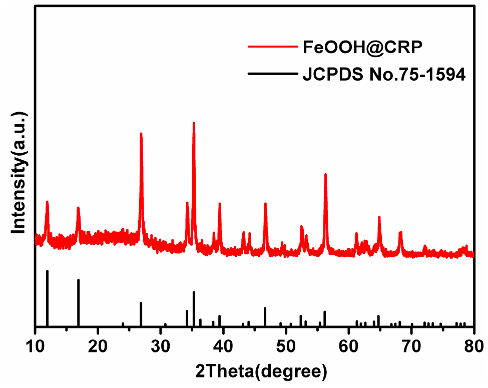

Figure S2. XRD pattern of FeOOH@C composite and the standard XRD pattern of FeOOH.

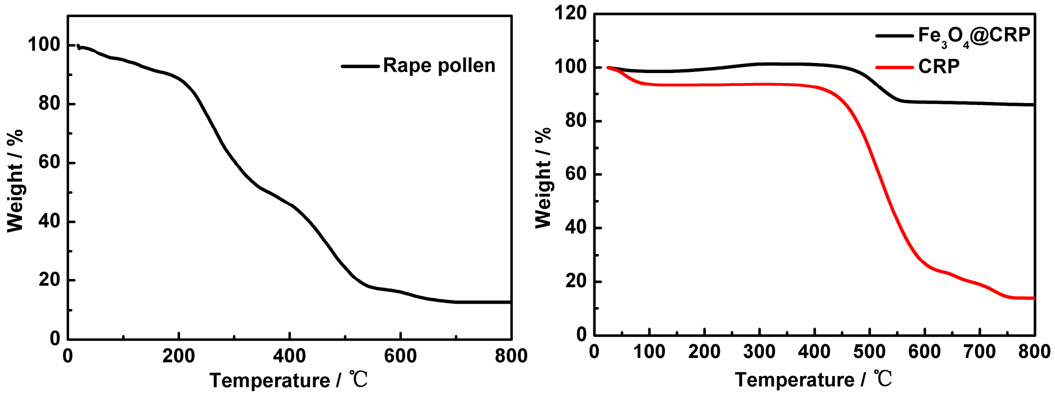

Figure S3. TGA curves of (**a**) rape pollen recorded in N_2, (**b**) C and Fe_3O_4@C composite recorded in air.

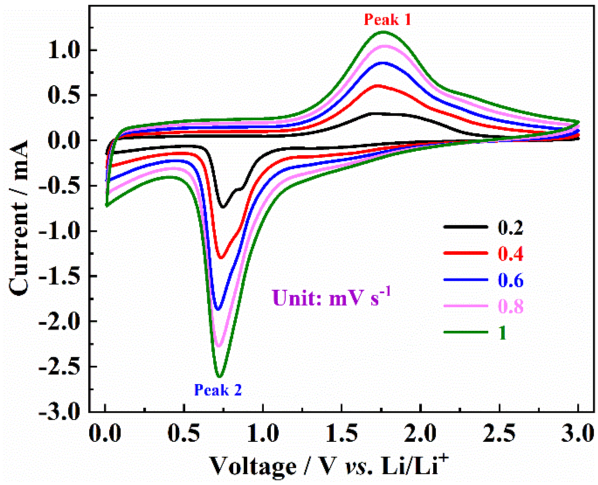

Figure S4. CV curves of Fe_3O_4@C electrode at different scan rates from 0.2 to 1 mV s^{-1}.

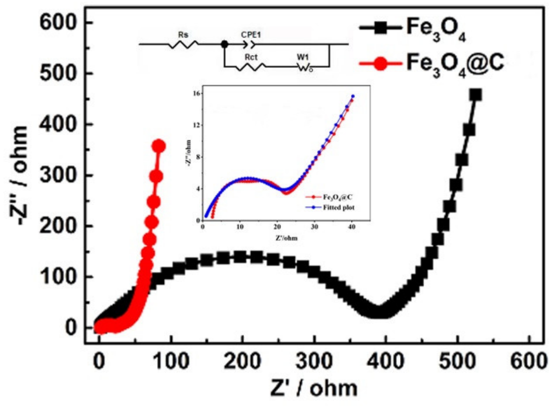

Figure S5. Nyquist plots of Fe_3O_4 and Fe_3O_4@C electrodes. Inset: Enlarged plot of high frequency range and the equivalent electrical circuit used to fit the experimental impedance spectra of Fe_3O_4@C electrode.

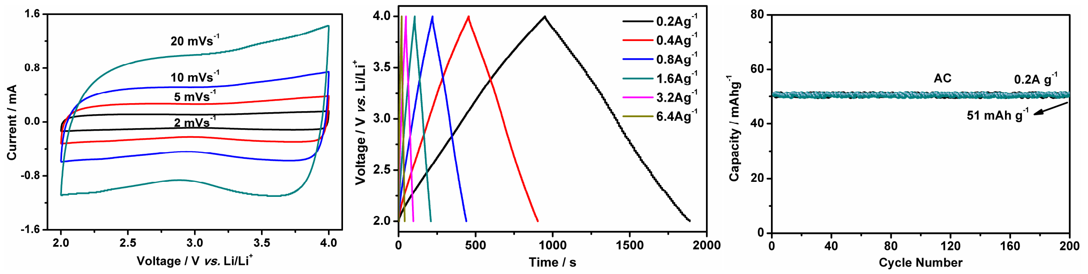

Figure S6. (**a**) CV curves of AC electrode at different scan rates from 2 to 20 mV s^{-1}. (**b**) GCD profiles at different current densities of 0.2 to 6.4 A g^{-1}. (**c**) The cycling performance of AC at 0.2 A g^{-1} between 2.0 and 4.0V.

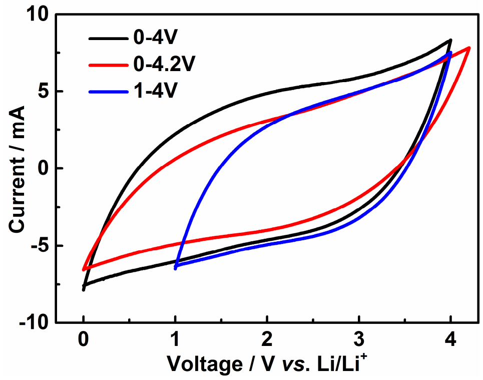

Figure S7. The CV curves of Fe_3O_4@C//AC LIC in different voltage ranges at a scan rate of 100 mV s^{-1}.

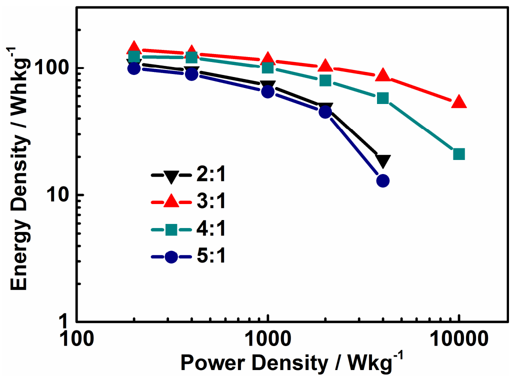

Figure S8. Ragone plots of the Fe_3O_4@C//AC LIC with mass ratios from 1:2 to 1:5.
